# Parkinson’s Disease: From Metabolism to Genetics—A Comprehensive Review

**DOI:** 10.3390/cimb48030254

**Published:** 2026-02-26

**Authors:** Cauan Duarte, Edislane Barreiros de Souza, João Rafael Dias-Pinto, Rodrigo Pinheiro Araldi

**Affiliations:** 1 Post-Graduation Program in Endocrinology and Metabolism, Paulista School of Medicine, Federal University of São Paulo (EPM-UNIFESP), São Paulo 04021-001, Brazil; c.duarte@unesp.br (C.D.); joao.dias@biodecisionanalitycs.com (J.R.D.-P.); 2 Faculty of Science and Letters, São Paulo State University (UNESP), Assis Campus, Assis 01049-010, São Paulo, Brazil; edislane.souza@unesp.br; 3 BioDecision Analytics, São Paulo 01451-917, Brazil

**Keywords:** Parkinson’s disease, mitochondrial dysfunction, oxidative stress, neuroinflammation, α-synuclein, RNA-Seq, bioinformatics

## Abstract

Parkinson’s disease (PD) is a progressive neurodegenerative disorder in which metabolic, inflammatory and proteostatic disturbances converge to drive dopaminergic neuron loss and widespread network failure. In this narrative review, we synthesize clinical, epidemiological and experimental evidence to organize PD pathophysiology around three interconnected metabolic axes: mitochondrial dysfunction and impaired glucose and lipid metabolism; chronic oxidative stress; and glial reprogramming and neuroinflammation, with α-synuclein acting as a central integrator at their interface. We then map how currently available dopaminergic, neuromodulatory and rehabilitative therapies interact with these axes, largely providing downstream symptomatic compensation while leaving core metabolic and inflammatory drivers only partially addressed. Next, we review RNA sequencing (RNA-Seq) and related transcriptomic studies in human brain and peripheral tissues, highlighting convergent differentially expressed genes in mitochondrial, synaptic, immune and proteostasis pathways, as well as major methodological challenges and opportunities for molecular subtyping and biomarker discovery. Together, these lines of evidence support a systems-level view of PD in which α-synuclein–centered metabolic failure and glial dysregulation are key therapeutic targets and in which high-quality RNA-Seq, integrated with advanced bioinformatics, may help define biologically grounded PD endotypes and accelerate the development of truly disease-modifying interventions.

## 1. Introduction

In 1817, the British physician James Parkinson published the seminal essay An Essay on the Shaking Palsy, in which he meticulously described six patients with involuntary tremor, muscular rigidity, and slowness of movement [1]. Although etiological understanding was nonexistent at the time, that clinical account inaugurated the characterization of one of the most studied neurological diseases in the world. Decades later, the term “Parkinson’s disease” (PD) became established, and advances in neuropathology allowed the illness to be associated with the progressive degeneration of dopaminergic neurons located in the Substantia nigra pars compacta (SNpc) and the consequent reduction in dopamine in the striatum [2].

However, PD is not confined to the SNpc. As the disease progresses, involvement extends to other brain regions, broadening network dysfunction and producing alterations in both motor and non-motor circuits of the central nervous system (CNS). Clinically, the disease is characterized by cardinal symptoms such as bradykinesia, rigidity, and resting tremor, as well as a broad non-motor spectrum, often prodromal, that includes hyposmia, constipation, REM sleep behavior disorder, depression/anxiety, fatigue, pain, orthostatic hypotension, and urinary and sexual dysfunctions, among others [1,2,3]. These manifestations may precede motor symptoms by years and, as the disease advances, substantially impacts quality of life. Despite the availability of dopaminergic therapies that provide substantial symptomatic relief, no disease-modifying treatment capable of halting or reversing neurodegeneration is currently available, underscoring the need for deeper mechanistic insights.

Moreover, the pathophysiology of PD involves a complex interplay of genetic, epigenetic, environmental, and metabolic factors. Although most cases are idiopathic, approximately 5% have a monogenic origin, involving mutations in genes such as *SNCA*, *LRRK2*, *PARK2*, *PARK7*, and *PINK1*, while 10–15% are attributed to polygenic inheritance [4,5,6,7]. Aging remains the strongest risk factor, and cumulative exposure to environmental insults—including pesticides, organic solvents, heavy metals, and air pollution—appears to interact with genetic susceptibility to trigger disease onset. At the mechanistic level, converging evidence points to misfolding and aggregation of α-Synuclein (α-Syn), mitochondrial dysfunction, oxidative stress, impaired autophagy-lysosomal pathways, synaptic failure, and chronic neuroinflammation as central processes driving nigrostriatal degeneration [4,5].

PD is currently recognized as the second most prevalent neurodegenerative disease worldwide, after Alzheimer’s disease (AD). According to estimates from the Global Burden of Disease (GBD) study, approximately 11.77 million individuals were living with the condition in 2021, reflecting a 274% increase since 1990. Forecasts suggest that by 2050, this number could rise to 25.2 million, driven predominantly by population aging (accounting for 89% of the increase), followed by population growth (20%) and changes in disease prevalence (3%) [2,3,8,9,10,11].

Epidemiological studies conducted in Latin America align with global trends in the rising burden of PD, reporting an average prevalence of 472 cases per 100,000 inhabitants (95% CI: 271–820) and an incidence of approximately 31 cases per 100,000 person-years [12,13,14]. Age-stratified analyses reveal a marked increase in prevalence among individuals aged over 80 years, reaching approximately 2079 cases per 100,000. These findings underscore population aging as the predominant factor driving the regional escalation of PD and emphasize the urgent need for public health strategies centered on early diagnosis, integrated care, and sustained support for an increasingly older and vulnerable population [8,10,11].

In Brazil, the Longitudinal Study of the Health of Brazilian Older Adults (ELSI-Brasil) cohort study estimated that the prevalence among individuals aged 50 and over is 0.84% (95% CI 0.64–1.09), with a projected growth from approximately 536,000 cases in 2024 to up to 1.25 million in 2060 [13]. These data reflect a growing challenge for healthcare systems, not only in terms of diagnosis and treatment but also of social and economic support, given the disabling and progressive impact of the disease [15].

Beyond the epidemiological impact, PD imposes a substantial and growing economic burden. In more recent studies, the average annual cost per person with PD in Brazil was estimated at US$4000, with 63% being direct costs (such as consultations, medications, and examinations) and 36% being indirect costs (such as loss of productivity and family burden) [16]. Globally, systematic reviews indicate that total costs per patient range from US$24,250 to nearly US$116,000 per year, depending on the stage of the disease and the region, with healthcare services accounting for about 46.1% of the costs, followed by loss of productivity (37.4%) and out-of-pocket expenses borne by patients and families (16.4%). These figures show that the economic burden of PD transcends the public healthcare system and deeply affects the socioeconomic structure of the family and society. In low- and middle-income countries, such as Brazil, unequal access to specialists and advanced therapies exacerbates disparities in disease management, contributing to late diagnoses and worse functional outcomes [16,17,18].

Although experimental and clinical studies have already identified several metabolic and molecular alterations in PD, the integration of these findings from a systems perspective remains limited. High-throughput RNA sequencing (RNA-Seq) technologies, combined with advanced bioinformatic pipelines, offer a powerful and unbiased approach to characterize gene expression networks, infer dysregulated pathways, and link molecular signatures to clinical and neuropathological phenotypes. In this context, in the present work we will discuss current evidence on the metabolic underpinnings of PD and the role of α-Syn as a central protein. In addition, we will highlight how RNA-Seq based approaches, integrated with modern bioinformatics, are redefining our understanding of PD pathophysiology and opening new avenues for biomarker discovery and the development of therapeutic strategies.

## 2. Methods

We conducted a comprehensive narrative review searched two databases (PubMed/MEDLINE and Scopus) using combined terms related to Parkinson’s disease and translational mechanistic axes (α-synuclein/SNCA, mitochondrial dysfunction/OXPHOS and oxidative stress, glial inflammation/microglia–astrocyte crosstalk, and lysosome–autophagy/mitophagy), as well as transcriptomics (RNA-seq, single-cell, single-nucleus), therapies (levodopa, adjunct therapies, deep brain stimulation, focused ultrasound, rehabilitation), and bioinformatics. The primary time window was 2020–2025; earlier “classic” studies were retained when essential for contextualization.

All data processing and visualization were performed in R v.4.5.2 using the packages dplyr, purrr, stringr, tibble, openxlsx, tidyr, and ggplot2.

## 3. Core Pathophysiology: Linking α-Syn Pathology to Metabolic Disruption

PD is a progressive neurodegenerative disorder caused by the accumulation of misfolded α-Syn, a presynaptic neuronal protein encoded by the *SNCA* gene, which plays a central role in disease onset and progression [19,20].

Under physiological conditions, α-Syn is involved in synaptic vesicle trafficking and neurotransmitter release. However, in PD, it undergoes conformational changes from its native α-helical structure to β-sheet-rich oligomers and fibrils that aggregate into Lewy bodies and Lewy neurites [21]. These toxic aggregates disrupt mitochondrial function, impair lysosomal degradation pathways, and trigger neuroinflammatory responses through microglial activation, ultimately leading to dopaminergic neuronal death in the SNpc [21,22,23]. These pathological processes are not limited to neurons, since glial cells also internalize α-Syn aggregates, which can either mitigate or exacerbate neurodegeneration depending on their activation state [24,25,26].

Metabolic dysfunction is a central feature of PD, with evidence pointing to impaired glucose metabolism, mitochondrial bioenergetics, and oxidative phosphorylation as key contributors to disease progression [21,22,23]. Alterations in glycolysis, the tricarboxylic acid (TCA) cycle, and the pentose phosphate pathway have been observed in PD patients, indicating widespread disruption of cellular energy homeostasis [19,20]. These metabolic deficits are closely linked to α-Syn toxicity, which interferes with mitochondrial dynamics and promotes the generation of reactive oxygen species (ROS) [23,24].

Mitochondrial dysfunction in PD is characterized by impaired electron transport chain activity, particularly in Complex I and IV, leading to reduced adenosine triphosphate (ATP) synthesis and increased oxidative stress [19,20]. α-Syn aggregates interact with mitochondrial membranes, disrupting their integrity and promoting the release of pro-apoptotic factors [21,23]. This mitochondrial damage is further exacerbated by iron accumulation and impaired mitophagy, which hinder the clearance of dysfunctional organelles [24,26].

Lipid metabolism also plays a critical role in PD pathogenesis, as α-Syn preferentially binds to specific lipid species, influencing its aggregation and membrane interactions [19,20]. Dysregulated cholesterol and sphingolipid metabolism affect lysosomal function and autophagy, impairing the degradation of α-Syn and contributing to its accumulation [21,23]. These lipidomic alterations have been proposed as potential biomarkers for early diagnosis and therapeutic targets for intervention [24,25].

In summary, the interplay between α-Syn pathology and metabolic dysfunction forms a vicious cycle that drives neurodegeneration in PD [19,20]. Therapeutic strategies aimed at restoring metabolic balance—such as enhancing mitochondrial function, modulating lipid metabolism, and reducing oxidative stress—hold promise for slowing.

## 4. Metabolic Dysfunctions in PD Pathophysiology

### 4.1. Mitochondrial Dysfunction in PD Pathophysiology

Mitochondrial dysfunction is central to PD, as the CNS depends heavily on OXPHOS. Failure compromises ATP production and affects particularly vulnerable regions, such as the dopaminergic neurons of the SNpc. The mitochondrial respiratory chain requires the integrity and coordinated functioning of its four complexes [27]. In these locations, electrons can escape and react prematurely with molecular oxygen, forming superoxide radicals [28,29]. In dopaminergic neurons, the high basal rate of mitochondrial respiration, combined with intense ROS production from dopamine metabolism, contributes to a greater vulnerability to oxidative stress. The failure of complexes I and III to efficiently retain electrons intensifies the imbalance between ROS production and antioxidant capacity, favoring persistent oxidative stress, reduced ATP synthesis, and ultimately culminating in cell death along the nigrostriatal pathway [30].

Complex I (NADH-coenzyme Q oxidoreductase): Complex I is the largest and first component of the respiratory chain, catalyzing the transfer of electrons from NADH to ubiquinone (coenzyme Q). During this reaction, protons are pumped from the mitochondrial matrix to the intermembrane space, contributing to the establishment of the proton electrochemical gradient necessary for ATP synthesis. Dysfunctions in complex I are among the main sources of electron leakage, favoring the production of ROS and being strongly implicated in the pathophysiology of neurodegenerative diseases, such as PD, particularly through selective vulnerability of dopaminergic neurons in the SNpc [31,32].

Complex II (succinate-ubiquinone oxidoreductase): Also known as succinate dehydrogenase, complex II integrates both the Krebs cycle and the electron transport chain. It catalyzes the oxidation of succinate to fumarate, transferring electrons directly to ubiquinone. Unlike the other complexes, complex II does not pump protons across the membrane, but it contributes to maintaining the electron flow in the chain, being crucial for the energy metabolism of cells [31,32].

Complex III (ubiquinone-cytochrome c oxidoreductase): Complex III, also called the cytochrome bc1 complex, is responsible for the transfer of electrons from ubiquinol (the reduced form of ubiquinone) to cytochrome c. This process is coupled with the pumping of protons into the intermembrane space, amplifying the proton gradient. Complex III is another critical point for possible electron leakage, especially in situations of oxidative stress, and can contribute significantly to ROS formation [33,34], thereby exacerbating oxidative damage in vulnerable neuronal populations such as nigrostriatal dopaminergic neurons in PD [31,32].

Complex IV (cytochrome c oxidase—COXCyt c): Complex IV represents the final step of the respiratory chain. It receives electrons from cytochrome c and transfers them to molecular oxygen, forming water. This process is essential for the maintenance of the electron transport chain and for the generation of the proton gradient, in addition to being coupled to the pumping of protons into the intermembrane space. The efficiency of complex IV is vital for cellular respiration, and its inhibition results in a severe energy deficit and the accumulation of toxic intermediates [31,32].

Aging induces a cascade of cellular and molecular changes that progressively compromise biological systems, with the brain being particularly vulnerable due to its high metabolic demands [27,35,36]. One of the most critical challenges in the aging brain is the efficient utilization of glucose, the primary energy substrate, which becomes impaired due to mitochondrial decline and oxidative stress [37,38,39]. Mitochondrial inefficiency leads to respiratory chain dysfunction, reduced ATP synthesis, and elevated reactive oxygen species (ROS) [40,41,42].

Under normal physiological conditions, the brain consumes approximately 20–25% of circulating glucose. Glucose transport into the brain is tightly regulated, primarily mediated by glucose transporter type 1 (GLUT1) in the blood–brain barrier (BBB) [37,38,39]. Once inside the brain parenchyma, astrocytes utilize GLUT1 and metabolize glucose via glycolysis, supporting the astrocyte-neuron lactate shuttle, which provides neurons with an auxiliary fuel source during heightened energy demand [33,43,44,45]. Neurons rely on the high-affinity glucose transporter GLUT3 to ensure efficient glucose uptake, even under low extracellular concentrations. Once internalized, glucose undergoes glycolysis to produce pyruvate, which enters the TCA (Krebs) cycle and fuels OXPHOS in mitochondria, resulting in robust ATP generation. [27,33,37,41].

With aging, this system undergoes progressive changes, including a reduction in the expression of GLUT1 at the BBB and GLUT3 in neurons, as well as the emergence of cerebral insulin resistance, further compromising glucose uptake and metabolism in nervous tissue. Such changes result in an energy deficit, greater production of reactive oxygen species, and, consequently, increased neuronal vulnerability, especially of dopaminergic neurons in the SNpc. In neurodegenerative diseases like PD, neuroimaging exams with FDG-PET show glycolytic hypometabolism in affected brain regions, reinforcing the central role of alterations in glucose uptake and metabolism in the pathophysiology of these diseases [46,47].

Together, these findings establish a mechanistic link between aging-related metabolic decline and the neurodegenerative cascade in PD, highlighting mitochondrial and glucose metabolism as promising therapeutic targets [27,35,36].

Taken together, these mitochondrial abnormalities, particularly chronic electron leakage at complexes I and III and sustained ROS generation in dopaminergic neurons, establish a persistent state of oxidative stress and bioenergetic failure that preferentially affects the nigrostriatal pathway, thereby consolidating mitochondrial dysfunction as a core driver of PD neurodegeneration [28,29,30,42].

Clinical and experimental evidence reinforces the relevance of mitochondrial dysfunction in the pathophysiology of PD. Pioneering studies demonstrated a significant reduction in mitochondrial complex I activity in the SNpc of PD patients [28,40,41,47,48,49]. Furthermore, animal models treated with selective complex I inhibitors, such as MPTP and rotenone, faithfully reproduce the clinical and neuropathological features of the disease, including the loss of dopaminergic neurons and the accumulation of α-Syn [50,51].

Mutations in genes that encode proteins essential for maintaining mitochondrial quality, such as *PINK1* and *Parkin*, are associated with familial forms of PD. These proteins regulate fundamental processes of mitochondrial dynamics: Fission, which allows for the elimination and redistribution of damaged mitochondria; fusion, which promotes the exchange of contents and the distribution of energy; and mitophagy, responsible for the selective removal of dysfunctional mitochondria, preventing the accumulation of organelles that could increase ROS production and compromise cellular metabolism. These mechanisms are crucial for preserving the viability and functionality of dopaminergic neurons [28,47,52].

Beyond its well-known role at presynaptic terminals, α-Syn is increasingly recognized as a key integrator at the interface between proteostasis, lipid homeostasis, and mitochondrial metabolism. Oligomeric and fibrillar α-Syn species localize to mitochondrial membranes, where they bind cardiolipin, inhibit complex I activity, disrupt mitochondrial membrane potential and calcium handling, and favor excessive mitochondrial fragmentation. In parallel, α-Syn aggregation in presynaptic terminals impairs synaptic vesicle recycling and increases cytosolic dopamine levels, which are readily oxidized, generating additional ROS and quinones that further damage mitochondrial proteins and DNA. Through these converging mechanisms, α-Syn acts as an upstream amplifier of bioenergetic failure and oxidative stress in dopaminergic neurons, functionally linking synaptic pathology to metabolic collapse in PD [46,52,53].

### 4.2. Oxidative Stress

Oxidative stress represents a central downstream mechanism in PD because, although ROS are essential for cellular homeostasis under physiological conditions, such as in redox signaling and host defense against pathogens, excessive production or failure of antioxidant mechanisms destabilizes this system and can have severe consequences, promoting oxidative attacks on lipids, proteins, and cellular DNA, leading to structural and functional tissue impairments, activating the inflammatory response and, consequently, resulting in cell death [54,55]. Five major ROS have been well characterized as contributors to PD pathophysiology, as summarized in Table 1.

Mechanistically, pathogenic α-Syn species disrupt mitochondrial homeostasis by impairing protein import, depressing electron transport (notably at complexes I and III), and favoring permeability-transition events; these defects increase electron leak to O_2_ and amplify superoxide (O_2_•−) generation, initiating a self-reinforcing redox loop in which ROS further oxidizes and aggregates α-Syn [59]. In parallel, extracellular α-Syn can activate microglia and NOX2, adding an inflammatory source of ROS to the neuronal burden [60].

Concurrently, in dopaminergic neurons, dopamine (DA) catabolism and auto-oxidation contribute additional oxidative load. Enzymatic deamination of DA produces H_2_O_2_, and DA-derived quinones react with mitochondrial and synaptic proteins (including α-Syn), stabilizing toxic conformers and further sustaining ROS production; iron-rich nigral microenvironments potentiate these redox reactions [59,60].

Accordingly, brain cells deploy coordinated antioxidant systems—superoxide dismutases (SOD1 cytosolic, SOD2 mitochondrial, SOD3 extracellular), catalase (peroxisomal), and glutathione peroxidases (GPX; cytosolic/mitochondrial)—to detoxify ROS and maintain redox homeostasis. SODs dismutate O_2_•− to H_2_O_2_, which is then reduced to H_2_O by catalase or GPX using reduced glutathione (GSH), with concomitant formation of GSSG [61]. In PD, these defenses are compromised: mitochondrial SOD2 activity can be functionally overwhelmed by persistent electron-transport defects; GSH levels fall in vulnerable regions, limiting GPX capacity; and peroxisomal detoxification may be insufficient to buffer sustained H_2_O_2_ flux. The net result is an oxidative milieu that feeds back into α-Syn misfolding, dopaminergic vulnerability, and progressive neurodegeneration [59,60,61]. Excess ROS also engages microglia and astrocytes, creating a feed-forward loop of oxidative damage and neuroinflammation that will be explored in detail in the next section.

In this redox context, glial cells—particularly astrocytes and microglia—emerge as key modulators of neuronal vulnerability [33]. Under physiological conditions, astrocytes supply GSH and antioxidant enzymes and help clear glutamate, while microglia contribute to debris removal and controlled ROS signaling, keeping oxidative stress within a regulated range [34,61]. In PD, however, excess ROS originating from mitochondrial dysfunction and dopamine metabolism acts as an initial trigger for sustained microglial activation, which in turn pushes astrocytes toward neurotoxic A1 states and aggravates failures in metabolic and antioxidant support [62,63]. As a result, a self-perpetuating cycle arises—ROS → M1/A1 glial activation → more ROS and cytokines → greater mitochondrial dysfunction and α-Syn aggregation → renewed glial activation—ultimately culminating in dopaminergic neuron loss in the nigrostriatal pathway [60,64].

### 4.3. Involvement of Glia in Energy Metabolism

In PD, glial cells—particularly astrocytes and microglia—play a central role in shaping brain energy metabolism and neuronal resilience. Among them, astrocytes are key to maintaining brain homeostasis, as they sustain energy metabolism through glucose uptake via GLUT1, glycolysis, and the lactate shuttle, which supplies neurons, particularly in regions of high synaptic activity [33,34]. Moreover, they provide antioxidant defense: astrocytes are the main source of GSH for neurons and other glial cells and harbor enzymatic systems such as GPX and SOD [61]. In addition, they regulate the synaptic microenvironment by taking up and recycling glutamate, thereby preventing excitotoxicity and buffering ionic fluctuations that could destabilize synaptic transmission [34]. At synaptic terminals, physiological α-Syn also participates in vesicle trafficking and neurotransmitter release, processes that are tightly coupled to local energy supply and glial metabolic support. Together, these functions maintain redox balance and protect neural tissue from the oxidative cost of a constantly active brain [34,61,62].

However, under chronic oxidative stress, inflammation, aging, or injury, this protective capacity becomes overwhelmed. As a result, astrocytes reduce the supply of GSH, show reduced GPX/SOD-mediated detoxification capacity, and lose efficiency in glutamate uptake and recycling, changes that favor excitotoxicity, neuronal Ca^2+^ overload, and mitochondrial ROS production [62,63]. In this setting, glial cells are not merely targets but also sources of ROS: microglia in a persistently activated state release pro-oxidant and pro-inflammatory mediators, amplifying oxidative damage and promoting neuronal death [62,63].

In the context of PD, excess ROS originating from neuronal mitochondria and dopamine metabolism acts as a trigger for sustained microglial activation [2,60,64]. Consequently, pro-inflammatory (M1) microglia release TNF-α, IL-1β, IL-6, and NO via iNOS; subsequently, NO reacts with superoxide (O_2_^−^) to generate peroxynitrite (ONOO^−^), a potent nitrating/oxidizing agent that damages lipids, proteins, and DNA, further impairing mitochondria and increasing ROS production [65]. Concurrently, misfolded α-Syn engages TLR2/TLR4, activating NF-κB and NOX2, which feed back into the pro-oxidant, pro-inflammatory milieu. Downstream, this signaling drives the A1 (neurotoxic) astrocyte phenotype, reduces astrocytic GSH supply, and disrupts both the glutamate–glutamine cycle and the lactate shuttle, thereby compromising metabolic and antioxidant support to neurons [49,66].

Beyond its effects on neurons, α-Syn also directly shapes glial metabolism. Astrocytes exposed to oligomeric or fibrillar α-Syn show impaired mitochondrial respiration, a shift toward glycolysis, and a reduced capacity to sustain the astrocyte–neuron lactate shuttle, thereby limiting the energy buffer available to dopaminergic neurons. In microglia, internalized α-Syn acts as a danger signal that not only activates inflammatory pathways but also enhances NOX2 activity and mitochondrial ROS generation, reinforcing a pro-oxidant metabolic profile [67]. These α-Syn driven changes in glial bioenergetics weaken antioxidant defenses, destabilize glutamate homeostasis, and lower the threshold for excitotoxicity and mitochondrial collapse in the nigrostriatal pathway [68].

Altogether, a self-perpetuating loop ensues: ROS → glial activation (M1/A1) → more ROS and cytokines → greater mitochondrial dysfunction/α-Syn aggregation → renewed glial activation. Therefore, breaking this cycle—by reducing NOX2/iNOS activity, reprogramming glia toward M2/A2 phenotypes, or reinforcing antioxidant capacity (GSH)—has been proposed as a strategy to slow dopaminergic neurodegeneration [49].

In PD, microglia participate in a first axis centered on danger sensing and inflammasome control, where pattern-recognition pathways not only prime NOX2/iNOS but also engage the NLRP3 inflammasome, enabling IL-1β maturation and sustaining chronic signaling when resolution fails [65,69].

In parallel, microglia orchestrate proteostasis and clearance—that is, they maintain intracellular protein quality via lysosomal/autophagic degradation of cargo and, respectively, remove extracellular threats via phagocytosis. They engulf misfolded α-Syn and neuronal debris through TREM2 and LC3-associated phagocytosis (a form of uptake coupled to autophagy); however, when clearance fails, extracellular α-Syn persists, propagating pathology and re-activating PRRs [64,65,68].

Metabolically, microglia in PD undergo a characteristic reprogramming. Pro-inflammatory states shift toward aerobic glycolysis with elevated pentose-phosphate flux, thus generating more NADPH to fuel NOX2, whereas pro-resolving states rely on mitochondrial OXPHOS and fatty-acid oxidation, thereby directly linking microglial bioenergetics to ROS output and to the persistence or resolution of neuroinflammation [65,69].

At the neurovascular interface, microglial mediators affect vascular integrity and iron handling. Chronic cytokine/ROS exposure disrupts BBB tight junctions and promotes iron accumulation in the SNpc, thereby amplifying oxidative chemistry and dopaminergic vulnerability [65,67].

Taken together, these complementary mechanisms, danger sensing/NLRP3, defective α-Syn clearance, complement-mediated synaptic pruning, metabolic reprogramming, and microglia-vasculature/iron crosstalk, outline multiple converging pathways through which microglia drive PD progression, while simultaneously highlighting therapeutic entry points (NLRP3 or complement modulation, TREM2 agonism, metabolic reprogramming) that align with strategies proposed to break this vicious cycle.

## 5. Comparative Analysis of Normal and PD States

Because metabolic processes are highly interconnected and can be challenging to follow without visual schematics, this section is intended to consolidate the core concepts needed to interpret the metabolic axes explored in this review, particularly in the context of PD. In Table 2, we present a succinct side-by-side comparison of normal metabolic function versus PD-associated metabolic remodeling, emphasizing the key nodes and triggers that underlie dysregulation.

Table 2 provides a systems-level comparison between physiological metabolic function and PD–associated dysregulation, organized into “interaction blocks” that capture interconnected molecular axes rather than isolated pathways. This framework illustrates how early metabolic disturbances—particularly those affecting mitochondrial bioenergetics and the tricarboxylic acid (TCA) cycle—can trigger cascading consequences across neurotransmission, redox balance, proteostasis, and immune–barrier mechanisms. By juxtaposing the healthy state with PD pathophysiology, the table underscores that PD is not driven by a single metabolic lesion, but rather reflects a coordinated breakdown of energy production, quality-control systems, and cellular homeostasis across vulnerable tissues and neural circuits.

Within the Metabolic change block, the table highlights a consistent energetic signature characterized by reduced oxidative phosphorylation efficiency and a compensatory shift toward glycolytic metabolism. Decreased ATP availability, together with an increased lactate/pyruvate ratio and reduced Complex I activity, supports a bioenergetic failure model in PD [50,70]. Notably, multiple TCA enzymes emerge as convergent metabolic bottlenecks (OGDH/KGDHC, SDH/Complex II, IDH3G, ACO2, and MDH2), indicating that impaired TCA flux is not a single-node defect but a distributed dysfunction across key catalytic steps. Collectively, these alterations constrain NADH supply and electron delivery to the respiratory chain, heighten susceptibility to oxidative stress, and favor accumulation of intermediary metabolites such as succinate, which may function as a pro-inflammatory signal [70,71,72,73,74,75,76]. In parallel, disruptions in glucose/insulin signaling and amino acid profiles suggest a greater reliance on alternative substrates and catabolic compensation, including reduced branched-chain amino acids (BCAAs) and increased ketone-related intermediates [37,77,78].

The Mitochondrial homeostasis block extends this interpretation by linking metabolic failure to impaired mitochondrial quality control and activation of pro-death signaling. Under healthy conditions, the balance between fission/fusion dynamics and mitophagy limits the accumulation of damage; in PD, reduced mitophagy efficiency (e.g., compromised PINK1/Parkin-mediated surveillance) permits the persistence of dysfunctional mitochondria, thereby increasing mtDNA damage and lowering cellular resilience [80,81]. This vulnerability is further amplified by stress-induced permeability transition events, in which mitochondrial membrane destabilization promotes cytochrome c release and caspase activation, ultimately driving apoptotic pathways [41,82]. Such mechanisms are particularly relevant for neuronal populations with high energetic requirements, especially those already exposed to substantial oxidative burden.

Neurotransmission & circuit vulnerability is represented by dopaminergic homeostasis and synaptic/axonal energetics, translating metabolic impairment into functional decline. The progressive loss of dopaminergic tone within the substantia nigra, accompanied by disturbances in dopamine synthesis and vesicular handling as well as increased MAO-B activity, supports the classical neurochemical phenotype of PD [70,83]. Importantly, the table also indicates that vulnerability may precede overt neuronal loss: ATP scarcity at synaptic terminals compromises neurotransmission and axonal transport, increases excitotoxic susceptibility, and destabilizes network function [84,85]. In this context, metabolic dysfunction should be viewed not merely as a downstream consequence of neurodegeneration, but also as a potential upstream driver of early circuit impairment.

Finally, the table integrates redox and metal/ion imbalance, impaired proteostasis, and activation of the immune–barrier axis as reinforcing loops that can sustain and amplify disease progression. Chronic oxidative and nitrosative stress—characterized by reduced GSH/NADPH availability and increased iNOS-mediated protein nitrosylation/nitration—damages membranes, proteins, and mitochondrial components, accelerating cellular instability [71,79,86,87]. Iron accumulation and Ca^2+^ overload further intensify this environment by promoting ROS generation, exacerbating endoplasmic reticulum stress, and weakening neuronal buffering capacity [71,88,89]. In parallel, dysfunction of the ubiquitin–proteasome system (UPS) and autophagy–lysosome clearance, including pathways associated with GBA/LRRK2 biology, reduces the removal of damaged proteins and organelles, thereby favoring α-synuclein accumulation and proteotoxic stress [70,90,91]. These molecular failures converge with neuroinflammation, systemic immune activation, and alterations in the gut–microbiota axis, reinforcing inflammatory signaling and BBB vulnerability and establishing a self-propagating feedback loop between central neurodegeneration and peripheral immunometabolic disturbance [50,92,93,94,95,96].

## 6. Pharmacological Therapies in Parkinson’s Disease: Integration with Pathophysiology

The therapies currently available for PD primarily focus on restoring nigrostriatal dopaminergic neurotransmission and modulating imbalanced excitatory pathways to alleviate motor symptoms; however, each pharmacological intervention acts at different points along the pathophysiological cascade, also influencing oxidative stress, neuroinflammation, and neuronal energy metabolism, as summarized in Table 3. Accordingly, from a mechanistic standpoint, these interventions can be categorized by their predominant pathophysiological target, which helps visualize how current strategies connect to the biochemical and cellular alterations of PD.

From a pathophysiological standpoint, most established therapies act relatively downstream in the degenerative cascade. Levodopa and dopamine agonists mainly compensate for striatal dopaminergic depletion, restoring basal ganglia output and improving motor function, but they do not directly correct mitochondrial dysfunction, chronic oxidative stress, or glial dysregulation [66,97,112]. Indeed, by increasing cytosolic dopamine and its oxidative metabolism, chronic dopaminergic stimulation may even add redox burden to susceptible neurons, partially overlapping with the metabolic and oxidative mechanisms discussed earlier [59,60]. Enzymatic inhibitors (MAO-B and COMT inhibitors) and adenosine A2A receptor antagonists act by prolonging or modulating dopaminergic and striatal signaling, again providing symptomatic relief without clearly modifying upstream metabolic failure or α-Syn-driven pathophysiology [98,99,100,106].

At the network level, device-aided interventions such as deep brain stimulation (DBS) and continuous dopaminergic delivery (LCIG, apomorphine infusion) remodel circuit dynamics within the cortex-basal ganglia-thalamus loops, reducing pathological oscillations and smoothing motor fluctuations [106,108]. Although these approaches can indirectly normalize activity-dependent metabolic demand and improve gait and quality of life, they still operate mainly at the level of circuit compensation rather than at the level of mitochondrial, redox, or glial biology [108]. By contrast, structured exercise and multimodal rehabilitation, while also symptomatic, have been associated with increased mitochondrial biogenesis, improved cerebral perfusion, and enhanced neurotrophic support, suggesting that non-pharmacological interventions may partially engage some of the metabolic axes highlighted in this review [111].

On this basis, an increasing number of potentially disease-modifying strategies now explicitly target metabolic and inflammatory nodes implicated in PD. Mitochondria-targeted antioxidants, modulators of PGC-1α and mitochondrial biogenesis, agents that enhance mitophagy (including those acting on the *PINK1/Parkin* pathway), and small molecules or antibodies designed to reduce α-Syn aggregation aim to interrupt the vicious cycle linking mitochondrial failure, excessive ROS production, and proteostatic stress [113,114]. Similarly, drugs targeting microglial activation, the NLRP3 inflammasome, or NOX2 activity seek to attenuate the glial amplification of oxidative and inflammatory damage, as discussed in the preceding sections [115]. To date, however, clinical results have been heterogeneous, underscoring the biological diversity of PD and the likelihood that only specific molecular subgroups will benefit from particular interventions [116].

Importantly, the repeated failure of several “mechanism-based” candidates in clinical trials does not necessarily invalidate the underlying biology but instead highlights recurrent translational bottlenecks [116]. First, many studies intervene after substantial nigrostriatal degeneration is already established, when reducing oxidative or inflammatory stress may be insufficient to reverse network-level dysfunction. Second, PD is biologically heterogeneous; enrolling unstratified likely dilutes benefits that may be confined to molecular subtypes with demonstrable mitochondrial impairment, α-syn pathology, or innate immune activation [116]. Third, negative trials are often difficult to interpret in the absence of robust CNS pharmacodynamic biomarkers: without evidence of target engagement (e.g., adequate brain penetration and measurable modulation of mitochondrial redox state or inflammasome-related readouts), lack of efficacy may reflect subtherapeutic exposure rather than an incorrect hypothesis [116].

Moreover, redox and neuroimmune pathways are highly adaptive; partial pathway blockade can trigger compensatory mechanisms, and broad antioxidant or anti-inflammatory approaches may fail to reach relevant cellular compartments (mitochondria, microglia) at tolerable doses. Finally, commonly used clinical endpoints and relatively short follow-up windows may be underpowered to detect disease modification, particularly without enrichment for faster progressors [117]. Collectively, these issues argue for earlier intervention, biomarker-driven patient stratification, and trial designs centered on demonstrable target engagement and mechanism-linked outcomes. Consequently, this suggests that single-target interventions may be insufficient for a multifactorial disorder, motivating combination strategies or stage-specific therapies aligned with each patient’s dominant pathogenic axis [117].

In light of these limitations of currently available therapies and the heterogeneity of treatment responses observed among patients, there is a clear need for tools capable of more directly connecting the metabolic and inflammatory mechanisms of PD with individual biological profiles and therapeutic outcomes. In this context, transcriptomic approaches, particularly RNA-Seq, offer an opportunity to profile gene expression networks, identify subgroups of patients with distinct metabolic signatures, and map patterns associated with more favorable or unfavorable treatment responses. In the next section, we will discuss how RNA-Seq, integrated with advanced bioinformatic pipelines, can be used to refine this understanding and guide the development of disease-modifying strategies that are more closely aligned with the underlying biology of PD.

## 7. RNA Sequencing (RNA-Seq)

Crucially, transcriptomics has not merely confirmed the metabolic model of PD; it has enabled its empirical testing, refinement, and its reassessment directly in human tissues. Metabolic frameworks predict that impaired OXPHOS, disturbed redox homeostasis, and defective mitochondrial quality control should leave a coordinated signature at the gene-network level. In line with these expectations, multiple bulk and single-cell/single-nucleus transcriptomic datasets report coordinated deregulation of nuclear-encoded mitochondrial programs—including respiratory chain assembly and NADH dehydrogenase modules—together with redox and detoxification pathways, mitochondrial dynamics, proteostasis and immune or glial activation signatures [118,119,120].

At the same time, RNA-Seq has revealed that these transcriptomic patterns are strongly context-dependent. First, downregulation of mitochondrial gene modules is often more pronounced in vulnerable dopaminergic neuronal populations than in bulk tissue analyses, highlighting the masking effects of cellular composition [121,122]. Second, some cohorts exhibit mixed or compensatory upregulation of stress-response and metabolic adaptation pathways—such as glycolytic rewiring and antioxidant programs—suggesting stage-specific or adaptive responses rather than a uniform decline in OXPHOS activity [118,119,120]. Third, peripheral transcriptomes (e.g., blood or PBMCs) frequently display inflammatory and metabolic signatures that do not trivially mirror those observed in brain tissue, underscoring systemic contributions to PD pathophysiology and limiting simplistic extrapolations across biological compartments [123,124,125].

Taken together, these findings indicate that “mitochondrial dysfunction” in PD is better conceptualized as a set of transcriptomically detectable, subtype- and cell-state-specific network perturbations that intersect with glial inflammation and proteostatic stress. Rather than a single uniform signal, transcriptomic profiles suggest context-dependent patterns shaped by cellular composition, disease stage, and tissue compartment, which helps explain why directionality and pathway-level readouts may vary across cohorts and experimental designs [120,126,127].

Beyond individual pathway analyses, RNA-Seq enables systems-level interrogation of PD biology by capturing coordinated expression shifts across mitochondrial bioenergetics, redox regulation, glial activation, and α-synuclein–related proteostasis, thereby linking the metabolic and inflammatory processes discussed above to molecular signatures observed in patient-derived tissues [128,129,130]. Given its breadth and sensitivity relative to probe-based platforms, RNA-Seq (including single-cell and single-nucleus strategies) has become a cornerstone for mapping context-dependent transcriptomic programs in PD [131,132,133].

**Figure 1:**
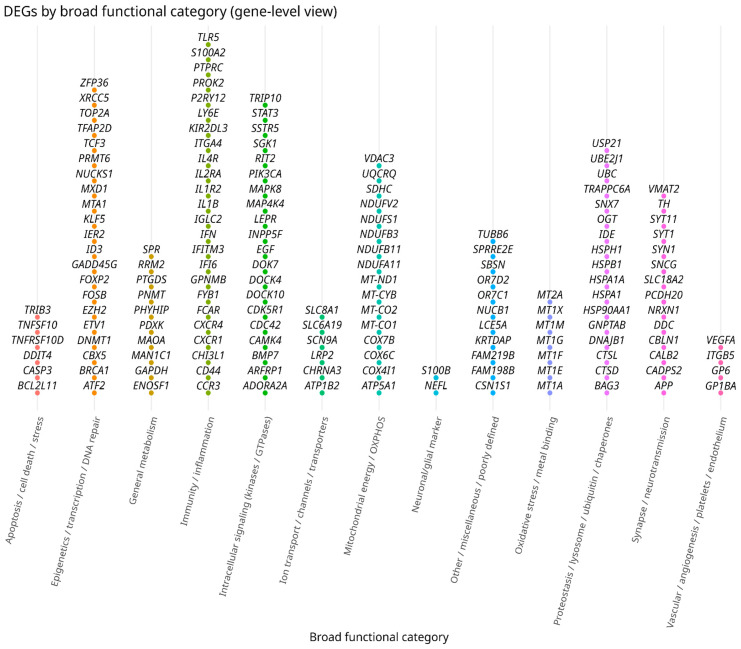
Distribution of some DEGs across macrofunctional categories in transcriptomic studies of PD. Each point represents one DEG identified in selected gene-expression studies of PD (listed in Table 3), labeled by its official gene symbol and grouped according to manually curated macrofunctional categories. The plot highlights the concentration of DEGs in pathways related to mitochondrial metabolism, synaptic function and neuroinflammation, reinforcing the mechanistic axes discussed throughout the review.

In PD research, RNA-Seq has been particularly informative for mapping coordinated pathway-level changes rather than isolated genes, while also supporting translational questions such as candidate-mechanism inference and biomarker prioritization [129,130,131,132,133,134]. To anchor these insights in the literature reviewed here, we summarize selected transcriptomic studies in Table 4 and integrate their reported DEGs into manually curated macrofunctional categories, highlighting convergence on mitochondrial, synaptic, and inflammatory modules (Figure 1).

**Figure 2:**
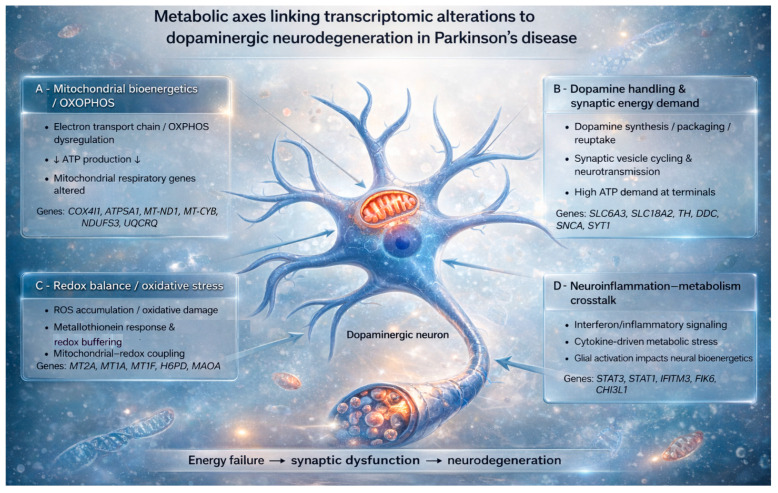
Metabolic axes linking transcriptomic alterations to dopaminergic neurodegeneration in PD. Schematic representation of a dopaminergic neuron highlighting interconnected metabolic and signaling axes identified from transcriptomic analyses. Block A illustrates mitochondrial bioenergetics and OXPHOS dysfunction, characterized by altered electron transport chain activity and reduced ATP production. Block B depicts dopamine handling and synaptic energy demand, emphasizing the high energetic cost of dopamine synthesis, vesicle cycling, and neurotransmission. Block C represents redox balance and oxidative stress, including ROS accumulation, metallothionein-mediated redox buffering, and mitochondria–redox coupling. Block D shows neuroinflammation–metabolism crosstalk, where inflammatory and interferon signaling, cytokine-driven metabolic stress, and glial activation impair neuronal bioenergetics. Arrows indicate causal and reinforcing interactions converging on energy failure, synaptic dysfunction, and progressive dopaminergic neurodegeneration. The figure was created by the authors and refined using an AI-assisted image-editing tool to improve clarity and readability; no scientific content was altered.

To integrate these transcriptomic findings into a unified biological framework, Figure 2 summarizes the major metabolic axes linking mitochondrial dysfunction, dopaminergic metabolism, redox imbalance and neuroinflammation in PD.

In addition to summarizing the differentially expressed genes (DEGs) reported across studies—acknowledging that each article applies its own analytical workflows, statistical criteria, and thresholds to define differential expression—we conducted a complementary, effect-size–harmonized analysis restricted to studies that provided complete Supplementary Materials with fold-change information. From each eligible dataset, we extracted gene-level log2 fold-change (log2FC) values (or converted reported fold-change metrics to a common log2FC scale when required) to enable a tissue-specific comparison across independently generated datasets. We then applied a standardized effect-size criterion (|log2FC| ≥ 0.40), classifying genes as upregulated (log2FC ≥ 0.40) or downregulated (log2FC ≤ −0.40) [128], and retained genes supported by at least three independent datasets within each tissue.

**Figure 3:**
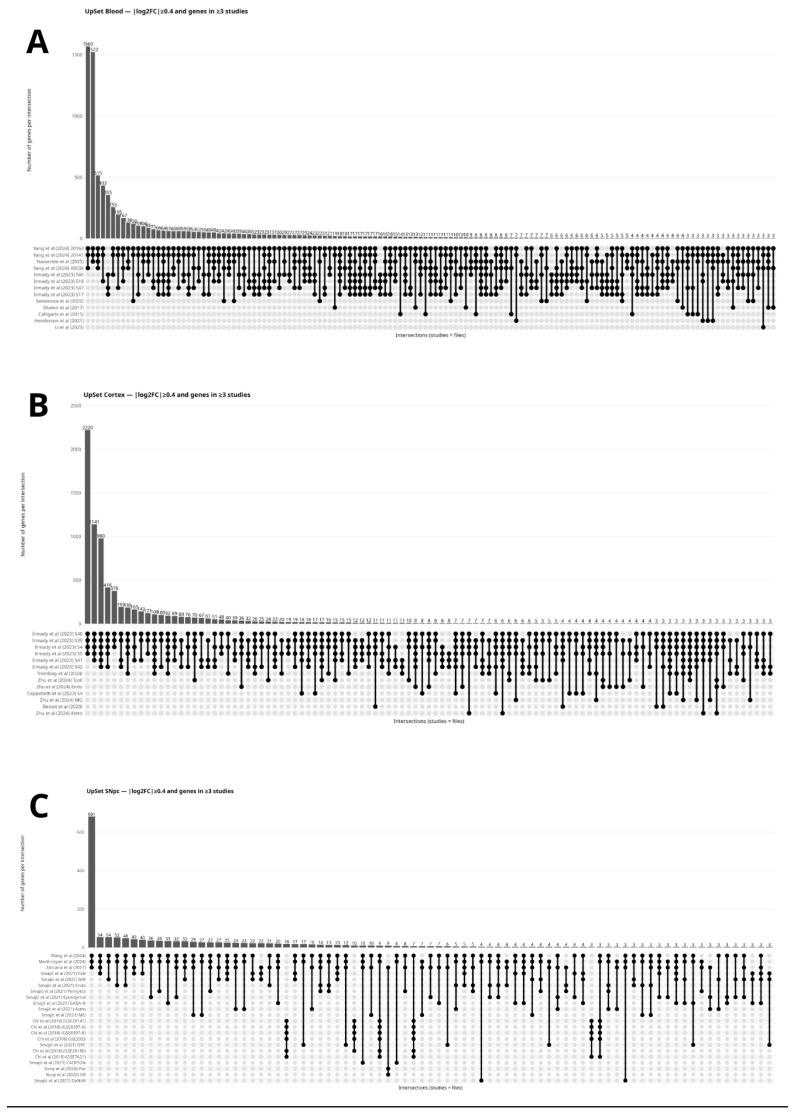
UpSet plots showing cross-study intersections of DEGs after effect-size harmonization (|log2FC| ≥ 0.40) and a minimum recurrence filter (genes present in ≥3 datasets), stratified by tissue. (**A**) Blood, (**B**) Cortex, and (**C**) SNpc. Each row in the lower panel represents one dataset (file/study), and each column represents a specific intersection of datasets. Connected dots indicate which datasets contribute to a given intersection, and the upper bars indicate the number of genes in that intersection. Only studies providing Supplementary Materials with fold-change values (or values convertible to log2FC) were included in this harmonized analysis. *Note:* when a single article provided multiple analyses (e.g., cell-type–specific subgroups), each was treated as an independent set for descriptive overlap visualization. A—Blood, B—Cortex and C—SNpc. References cited in Figure 3: [118,119,120,123,124,125,126,135,137,139,140,148,149,150,151,152,154,155].

To visualize cross-study overlap under this shared effect-size definition, we used intersection-based UpSet plots (Figure 3A–C). For each tissue (Blood, Cortex, and SNpc), each dataset (including cell-type–specific Supplementary Material when provided by a study) was treated as an independent set, and intersections represent genes jointly meeting the |log2FC| threshold in the corresponding combination of datasets. The bar heights quantify the number of genes per intersection, providing a compact summary of both replication breadth and the distribution of shared versus dataset-specific signals under a unified effect-size filter.

This study analyzed third-party data that are publicly available as Supplementary Materials of previously published articles (see References [118,119,120,123,124,125,126,135,137,139,140,148,149,150,151,152,154,155]). No new raw data were generated in this study. All gene-level results underlying Figure 3A–C are available in the Supplementary Material of the present manuscript. Supplementary Table S1 (“Harmonized log2 fold-change values for differentially expressed genes across tissues (S1)”) contains the harmonized quantitative log2FC values organized by tissue. The original datasets remain available from the Supplementary Materials of the respective publications and/or from the corresponding authors, subject to the terms of those publications.

The UpSet plots provide a compact view of reproducibility across datasets under a standardized effect-size threshold (|log2FC| ≥ 0.40) and a minimum recurrence requirement (≥3 datasets) [128]. Compared with a simple aggregated count by evidence range, this representation helps determine whether overlap is concentrated in a few study combinations (large intersections) or distributed across many smaller intersections, which may suggest greater heterogeneity across data sources.

In blood (Figure 3A), several intersections show relatively high bar heights, indicating that a substantial number of genes meet the effect-size threshold across multiple datasets simultaneously. At the same time, the presence of many smaller intersections (including several with low counts) suggests that part of the signal remains specific to subsets of studies, consistent with differences in cohort design, cellular composition, platforms, and analytical pipelines across peripheral datasets.

In cortex (Figure 3B), large intersections involve multiple datasets derived from the same study (e.g., different subgroups/strata). This is expected when distinct analyses are conducted from the same cohort and pipeline, reflecting high internal consistency under the standardized effect-size threshold. In this context, the larger intersections should be interpreted primarily as a portrait of agreement among these related analyses, while intersections that also include other studies help contextualize the extent to which the signal extends across independent sources.

In SNpc (Figure 3C), intersections tend to concentrate a moderate number of genes within specific dataset combinations, while many intersections remain small. This pattern is compatible with the higher variability expected in post-mortem tissue and differences in material availability, quality-control criteria, cellular composition, and normalization strategies across studies. Overall, under a shared effect-size threshold, a subset of genes appears recurrently across multiple datasets, but overlap remains partially fragmented, reflecting biological and methodological heterogeneity.

Importantly, these plots are not intended to adjudicate discrepancies between studies. Rather, they provide a descriptive, tissue-stratified summary of how frequently genes meet a shared effect-size threshold across independently generated datasets and how overlap patterns distribute across combinations of studies.

These cross-study patterns should be interpreted in light of methodological variability in RNA-seq workflows, which can influence DEGs detection and effect-size estimates. Despite recent advances, the analysis of RNA-Seq data continues to represent a significant methodological challenge, requiring rigor from the initial stages of sample preparation [158]. The specialized literature recommends attention to fundamental aspects, such as the efficient removal of ribosomal RNA, since it accounts for more than 90% of total cellular RNA and the assessment of RNA concentration and integrity, preferably with RNA Integrity Number (RIN) values greater than 6 [134]. Another critical point involves the choice of enrichment strategy: poly(A) selection is indicated for most mRNAs and many lncRNAs, whereas rRNA depletion may be more appropriate when the goal is to analyze non-polyadenylated transcripts or degraded samples, such as post-mortem brain tissue [134].

Subsequent steps include controlled fragmentation of RNA, necessary to adapt insert size to the sequencing platforms, and the definition of read type, which can be single-end (one read, therefore more economical) or paired-end (two reads, at roughly double the cost) [159]. In PD studies, these decisions acquire additional importance due to frequent constraints in sample availability and quality, particularly for substantia nigra and other vulnerable regions.

At the bioinformatic level, analysis pipelines typically span from quality assessment with tools such as FastQC [160] and MultiQC [161] tools, to the alignment of reads with the reference genome, with tools such as STAR [162], HISAT2 [163], or Bowtie2 [164] to the quantification and normalization of transcripts, using methods such as TPM [165] DESeq2 [166], or edgeR [167]. Each methodological decision directly impacts the interpretation of the results, and the choice of databases for functional enrichment analysis can even generate distinct predictions, highlighting the need for critical rigor in the analysis [128,133].

Despite these limitations, recent advances have incorporated artificial intelligence (AI) technologies and more robust statistical methods, with the aim of optimizing analysis pipelines and reducing technical biases [132]. In PD, such approaches are increasingly being used to define molecular subtypes characterized by specific combinations of mitochondrial, oxidative, glial, and synaptic signatures, and to relate these profiles to clinical phenotypes and treatment responses. In this way, the consolidation of good methodological practices, combined with the development of new analytical strategies, is essential to ensure that RNA-Seq continues to play a central role in elucidating PD pathophysiology, particularly the metabolic and inflammatory axes coupled to α-Syn and in identifying new therapeutic targets and biomarkers capable of supporting truly disease-modifying interventions.

## 8. Conclusions

In summary, this review sought to organize Parkinson’s disease pathophysiology around its central metabolic axes, highlighting the integrative role of α-synuclein at the interface between mitochondrial dysfunction, oxidative stress, glial inflammation, and proteostasis. Conformational alterations and pathological aggregation of α-synuclein may contribute to a self-reinforcing cascade involving respiratory chain impairment, sustained reactive species production, microglial activation, and strain on protein quality-control systems, consistent with a progressive neurodegenerative disorder that still lacks clearly disease-modifying therapies. Accordingly, currently available treatments remain largely focused on dopaminergic compensation and circuit modulation, providing important symptomatic relief while acting predominantly downstream of core degenerative mechanisms.

Against this background, integrative transcriptomic approaches offer a powerful strategy not merely to catalog differentially expressed genes, but to place them within coherent biological frameworks—linking signals to pathways and modules, and relating them to clinical, imaging, and treatment-response phenotypes. The convergence of RNA-Seq with advanced bioinformatics, network biology, and pharmacological screening resources may help prioritize molecular targets capable of modulating key disease-relevant programs.

Importantly, the evidence synthesized here also highlights a practical challenge for the field: substantial heterogeneity in the analytical workflows used to define differential expression. Differences in preprocessing, normalization, statistical modeling, thresholds, and cohort composition can yield variable DEG calls, including inconsistent directionality across datasets and limited cross-study comparability. These observations support the value of transparent, harmonized analytic pipelines and reporting standards that facilitate more interpretable and reproducible transcriptomic signatures across tissues and study designs.

Thus, the path toward translating DEG signals into robust biomarkers and therapeutic targets in PD—and potentially other neurodegenerative disorders—will likely depend on the integration of high-quality RNA-Seq data, harmonized analytical frameworks, and mechanistically informed interpretation of molecular findings.

## Figures and Tables

**Table 1:** Reactive Oxygen Species (ROS): Chemical Properties, Origins, and Roles in Parkinson’s Disease Pathophysiology Related to α-Synuclein Toxicity.

Ros Type	Chemical Formula	Primary Origin	Role in PD (α-Syn Context)	References
Superoxide	O_2_^−^	Mitochondrial electron leakage (Complexes I & III); dopamine auto-oxidation	- Promotes α-Syn aggregation - Impairs mitochondrial function - Initiates oxidative damage to lipids and proteins	[56,57,58]
Hydrogen Peroxide	H_2_O_2_	Superoxide dismutation via superoxide dismutase (SOD); peroxisomal metabolism	- Diffuses across membranes - Reacts with iron to form hydroxyl radicals - Exacerbates α-Syn toxicity and neuroinflammation	[56,57,58]
Hydroxyl Radical	OH•	Fenton reaction (H_2_O_2_ + Fe^2+^); lipid peroxidation	- Highly reactive - Causes DNA fragmentation, lipid peroxidation, and protein carbonylation - Accelerates α-Syn misfolding	[56,57,58]
Singlet Oxygen	^1^O_2_	Photochemical reactions; enzymatic processes; immune response	- Oxidized amino acids in α-Syn - Promotes cross-linking and aggregation - Contributes to Lewy body formation	[56,57,58]
Peroxynitrite	ONOO^−^	Reaction of superoxide with nitric oxide (NO); inflammation	Nitrates tyrosine residues in α-Syn Impairs mitochondrial enzymes Triggers apoptosis in dopaminergic neurons	[56,57,58]

**Table 2:** Comparative analysis of normal metabolic function and Parkinson’s disease–associated dysregulation.

Interaction Block	Biological Category	Physiological (Healthy) Condition	Parkinson’s Disease (PD) Pathophysiology	Key Metabolic Dysregulation	Reference
Metabolic change	ATP production	High ATP production via efficient OXPHOS.	Bioenergetic failure; shift from OXPHOS to anaerobic glycolysis (Warburg-like pattern).	- ↓ ATP - ↑ Lactate/ Pyruvate ratio - ↓ Complex I activity.	[50,70]
Metabolic change	α-Ketoglutarate Dehydrogenase (OGDH/KGDHC)	Rate-limiting TCA step; converts α-KG to succinyl-CoA; generates NADH.	↓ Activity (up to ~50%) in SNpc and cerebellum; TCA “bottleneck.”	- ↑ α-KG - ↑ ROS - ↓ NADH supply for Complex I.	[70,71]
Metabolic change	Succinate Dehydrogenase (SDH/Complex II)	Oxidizes succinate to fumarate; links TCA to ETC.	↓ Activity/ Expression; vulnerable to nitrosative modification (e.g., S-nitrosylation)	- ↓ Electron flow - ↑ Succinate (pro-inflammatory).	[72,73]
Metabolic change	Isocitrate Dehydrogenase (IDH3G)	Converts isocitrate to α-KG.	↓ Activity consistent with a metabolic “bottleneck.”	- ↓ α-KG supply - disrupted α-KG/Fumarate balance.	[70,74]
Metabolic change	Aconitase (ACO2)	Isomerizes citrate to isocitrate (early TCA flux).	↓ Activity due to oxidative inactivation.	- ↑ Sensitivity to O_2_•− - ↓ early TCA flux.	[71,75]
Metabolic change	Malate Dehydrogenase (MDH2)	Converts malate to oxaloacetate; supports citrate synthesis and TCA cycling.	↓ Expression contributes to TCA stalling.	- ↓ Oxaloacetate regeneration - ↓ citrate synthesis support.	[70,76]
Metabolic change	Glucose/Insulin Signaling	Normal insulin sensitivity; efficient neuronal glucose uptake.	Brain insulin resistance; association with metabolic syndrome traits.	- ↑ Insulin resistance - ↓ glucose flux into astrocytic TCA cycle.	[37,77]
Metabolic change	Amino Acid Profiles	Stable BCAA levels; balanced amino acid metabolism.	Shift toward amino acid catabolism for energy compensation.	- ↑ 3-hydroxybutyrate - ↓ plasma BCAAs - ↑ urea-cycle intermediates.	[77,78]
Metabolic change	Lipid Metabolism	Balanced FAO and lipid signaling; stable membrane lipid composition.	Lipid-droplet accumulation; altered phospholipid profiles.	- ↑ Triglycerides - ↓ FAO - ↑ lipid peroxidation (MDA).	[70,79]
Mitochondrial homeostasis	Mitochondrial dynamics & quality control (Mitophagy)	Balanced fission/fusion; damaged mitochondria removed by mitophagy.	Impaired quality control with accumulation of dysfunctional mitochondria.	- ↑ Dysfunctional mitochondria - ↓ Mitophagy (PINK1/Parkin) - ↑ mtDNA damage.	[80,81]
Mitochondrial homeostasis	Mitochondrial permeability transition / apoptosis	Mitochondrial membranes remain stable; pro-apoptotic factors are retained.	Stress triggers permeability transition and cell-death signaling.	- ↑ mPTP opening - ↑ cytochrome c release - ↑ caspase activation.	[41,82]
Neurotransmission & circuit vulnerability	Dopamine Homeostasis	Regulated dopamine synthesis; efficient vesicular storage.	Progressive loss of SNpc dopaminergic neurons; reduced dopaminergic tone.	- ↓ Dopamine - ↓ Tyrosine- to-Phenylalanine ratio - ↑ MAO-B activity.	[70,83]
Neurotransmission & circuit vulnerability	Synaptic/axonal energetics	Adequate ATP supports synaptic transmission and axonal transport.	Energy deficit at terminals/axons; transport defects; increased vulnerability.	- ↓ Synaptic ATP - ↓ axonal transport - ↑ excitotoxic vulnerability.	[84,85]
Redox & metal/ion homeostasis	Redox Balance	ROS controlled by antioxidant systems; GSH recycling supported by NADPH.	Persistent oxidative stress; gradual depletion of antioxidant capacity.	- ↑ ROS - ↓ GSH - ↓ NADPH (PPP).	[71,79]
Redox & metal/ion homeostasis	Nitrosative Stress	Controlled NO signaling for physiological functions.	Excess ONOO^−^ formation; protein nitrosylation/nitration.	- ↑ iNOS - ↑ 3-NT– modified proteins.	[86,87]
Redox & metal/ion homeostasis	Iron Metabolism	Tight iron storage (ferritin); low labile iron pool.	Selective iron accumulation in SNpc; impaired export.	- ↑ Labile Fe^2+^ - ↑ DMT - ↓ ferroportin 1	[71,88]
Redox & metal/ion homeostasis	Calcium Homeostasis	Precise buffering by mitochondria/ER; low cytosolic Ca^2+^.	Ca^2+^ overload; loss of buffering capacity; chronic ER stress.	- ↑ Cytosolic Ca^2+^ - ↑ Cav1.3 density - ↑ MCU oligomerization.	[88,89]
Proteostasis & clearance	Protein Proteostasis	UPS + autophagy-lysosome maintain proteome quality.	Impaired clearance; Lewy body formation; proteotoxic stress.	- ↑ α-Syn aggregation - ↓ lysosomal degradation.	[70,90]
Proteostasis & clearance	Autophagy–lysosome axis (GBA/LRRK2-related)	Efficient autophagy and lysosomal degradation of proteins/organelles.	Lysosomal dysfunction reduces clearance efficiency.	- ↓ Lysosomal activity - ↓ GCase (GBA) - ↑ α-Syn accumulation	[90,91]
Immune–barrier axis	Neuroinflammation	Mostly homeostatic (M2-like) microglia; neurotrophic support.	Chronic microglial activation (M1-like); reactive astrogliosis.	- ↑ TNF-α - ↑ IL-1β - ↑ IL-6 - ↑ NLRP3 activation.	[50,92]
Immune–barrier axis	Systemic Inflammation	Normal leukocyte ratios; low peripheral cytokines.	Systemic immune activation; BBB vulnerability/compromise.	- ↑ NLR - ↑ plasma IL-12B and OPG.	[93,94]
Immune–barrier axis	Gastrointestinal /Microbiota	Diverse microbiota; intact intestinal barrier; beneficial metabolites (SCFAs).	Dysbiosis; increased permeability (“leaky gut”); inflammatory signaling.	- ↓ SCFAs - ↑ pro-inflammatory bacterial metabolites.	[95,96]

**Table 3:** Pharmacological interventions in Parkinson’s disease organized by predominant pathophysiological targets.

Pathophysiological Target	Treatment	Use	Mechanism of Action	Main Benefits	Limitations (Non–Adverse Effects)	Common Adverse Effects	References
Dopamine replacement	Levodopa + carbidopa benserazide (oral)	Symptomatic gold standard	Dopamine precursor crosses BBB; peripheral dopa-decarboxylase inhibitor reduces peripheral effects	Greatest effect on motor symptoms; improves ADLs	Symptomatic control does not modify disease; efficacy fluctuates as disease progresses; multiple daily doses interaction with high-protein meals	- Nausea - Hypotension - Somnolence - Dyskinesias - ON/OFF fluctuations long term	[63,69]
Direct stimulation of dopaminergic receptors	Dopamine agonists (pramipexole, ropinirole, rotigotine)	Drugs that stimulate DA receptors	Direct D2/D3 stimulation (±others)	Reduces OFF time; “levodopa-sparing” early on	Lower potency than levodopa; worse cognitive tolerability in older adults; slow titration	- Somnolence - Edema - Nausea - Impulse-control disorders - Hallucinations	[67,97]
Reduction of central dopamine degradation	MAO-B inhibitors (rasagiline, selegiline, safinamide)	Oral adjuncts	Inhibit dopamine breakdown (safinamide: also glutamate modulation)	Increase ON time (safinamide with less troublesome dyskinesia)	Modest effect; interactions with MAOIs/SSRIs; no disease modification	- Insomnia - Nausea - Headache	[98,99]
Reduction of peripheral levodopa degradation	COMT inhibitors (entacapone, opicapone)	Adjuncts to levodopa	Prolong levodopa half-life	Reduce OFF; opicapone once daily	Benefit depends on levodopa; may increase dyskinesia; higher cost for newer agents	- Diarrhea - Amber urine - Increased dyskinesia	[100,101]
Glutamatergic modulation	Extended-release amantadine (Gocovri)	For dyskinesia and OFF	NMDA antagonism + indirect dopaminergic effects	Reduces dyskinesia and OFF	Effect may wane over time; dose adjustment in CKD; evening dosing	- Insomnia - Livedo reticularis - Edema - Confusion	[68,102]
Cholinergic modulation	Anticholinergics (trihexyphenidyl, benztropine)	Symptomatic for tremor	Muscarinic blockade	Useful for predominant tremor	Poorly tolerated >60 years; benefit largely limited to tremor	- Dry mouth - Constipation - Urinary retention - Confusion	[103,104]
Striatal adenosinergic modulation	A2A antagonist (istradefylline)	Oral adjunct	Blocks striatal A2A (indirect pathway)	Reduces OFF as levodopa add-on	Modest gain; cost; variable availability	- Dyskinesia - Insomnia - Nausea	[105]
Dopamine replacement	Levodopa/ carbidopa intestinal gel (LCIG/duodopa)	Device- aided therapy	Continuous jejunal infusion via PEG-J tube	Robust reduction in OFF and dyskinesia	Invasive; system maintenance (infection, tube issues); high costs	- Stoma/tube - Complications - Weight loss	[63,106]
Direct stimulation of dopaminergic receptors	Continuous subcutaneous apomorphine infusion	Dopamine agonist via pump	Continuous dopaminergic stimulation	Reduces OFF in advanced PD	Requires skin care and antiemetic in some countries; patient selection	- Nausea - Subcutaneous nodules - Hypotension - Somnolence	[67,107]
Circuit-level/surgical neuromodulation	Deep brain stimulation (DBS - STN/GPi)	Functional neurosurgery	Modulates basal-ganglia circuits	Large reduction in OFF and dyskinesia; QoL improvement	Restricted eligibility; requires programming and follow-up; may worsen speech/mood	- Surgical risks (hemorrhage/infection) - Speech/balance issues	[108]
Circuit-level/surgical neuromodulation	MRI-guided focused ultrasound (STN/GPi lesioning)	Thermal ablation without craniotomy	Unilateral focal lesion	Useful for asymmetric symptoms; alternative to DBS	Irreversible; typically unilateral; stringent selection	- Dysarthria - Paresthesias - Gait disturbances	[109,110]
Non-pharmacologic rehabilitation	Rehabilitation/structured exercise (aerobic, strength, balance, cueing)	Non-pharmacologic interventions	Network plasticity/compensation; improves gait and balance	Safe; improves function/UPDRS; impact on FOG with cueing	Effect depends on adherence and protocol; study heterogeneity	- Fatigue - Musculoskeletal pain	[111]

**Table 4:** Overview of gene-expression studies associated with PD, indicating the tissue source/brain region, number of controls/patients, cohort age range, and some differentially expressed genes (DEGs) reported in each study *.

Tissue Source/Brain	Control/Patients	Age Range	DEGs Identified	Reference
Blood	233/205	28–80	*COX4I1, ATP5A1, VDAC3, XRCC5, STAT3, USP21*	[123]
Blood	32/52	51–78	*CBX5, TCF3, MAN1C1, DOCK10*	[124]
Blood	3/3	49–65	*PTGDS, ADORA2A, MTA1*	[135]
Blood	4/4	44–69	*MT-CYB, MT-ND1, NDUFB3, UQCRQ, COX6C, CASP3*	[136]
Blood	13/11	45–73	*EGF, BRCA1, LEPR, APP*	[137]
Blood	9/10	55–78	*GP1BA, GP6, P2RY12, ITGB5*	[138]
Blood	16/46	60–90	*IFN, IFI6, IFITM3, IER2, LY6E, MT2A*	[139]
Blood	16/23	45–106	*CHI3L1, PROK2, FAM198B, ID3, MXD1*	[125]
Blood	15/15	63–78	*FOSB, COX7B, COX6C, TUBB6*	[140]
Blood	30/30	50–57	*CASP3, PIK3CA, VEGFA, IL2RA, IL4R, ITGA4, BCL2L11*	[141]
Blood	189/390	49–72	*DOCK4, TRIB3, SLC6A19*	[142]
Blood	594/691	44–73	*RIT2, INPP5F, MT-CO1, MT-CO2, NDUFB11, NDUFA11, GAPDH, SYT1*	[143]
Blood and Skin	12/12	50–80	*OGT, UBE2J1, KIR2DL3, LCE5A, ENOSF1, FAM219B, CTSL, CXCR1, SBSN, ETV1, SPRRE2E, CTSD, KRTDAP, S100A2*	[144]
Cerebrospinal Fluid	30/27	18–99	*DNMT1, EZH2, CCR3, SSTR5, PTPRC, UBC, NDUFV2, BMP7, SCN9A*	[145]
Cortex (BA9)	6/6	65–96	*MT1F, MT1G, CAMK4, HSPA1A*	[118]
Cortex (BA9)	80/60	62–84	*MT1A, MT1E, MT1F, MT1G, MT1M, MT1X, MT2A*	[119]
Cortex (BA9)	6/12	62–84	*GNPTAB, IDE, FOXP2, CDK5R1, NRXN1*	[146]
Cortex (BA9)	29/49	53–86	*DDIT4, SPR, TRIP10, TNFRSF10D, PRMT6, TLR5*	[147]
Cortex (BA8/9)	23/44	65–102	*SNX7, NUCB1, PDXK, PHYHIP, MAP4K4*	[148]
Frontal Lobe Biopsy	5/6	38–70	*CSN1S1, GADD45G, TFAP2D, SGK1, ZFP36, TNFSF10*	[149]
Olfactory	19/19	74–92	*CHRNA3, SNCG, OR7D2, TOP2A, RRM2, IGLC2*	[150]
Striatum + Blood	40/35	60–85	*CAMK4, FCAR, IL1R2, PROK2, TRAPPC6A, ARFRP1, NUCKS1*	[151]
Substancia Nigra + Blood	70/80	41–94	*NDUFS1, COX4I1, SDHC, CDC42, MAPK8*	[152]
Substancia Nigra + Putamen	10/10	65–86	*OR7C1, KLF5, IL1B, DOK7, PCDH20*	[153]
Substancia nigra	9/23	69–93	*HSPA1A, DNAJB1, BAG3, SYN1, CALB2, NEFL*	[134]
Substancia nigra	14/15	57–93	*SYT11, MT2A, MT1E, ATF2*	[126]
Substancia nigra	9/23	69–93	*MT2A, MT1E, HSPB1, HSPH1, HSPA1*	[154]
Substancia nigra	5/5	64–87	*LRP2, PNMT, CXCR4, MAOA, CBLN1*	[155]
Substancia nigra	6/8	68–88	*ATP1B2, SLC8A1, MT-CO1, MT-CO2, MT-CYB, FYB1*	[156]
Substancia nigra	5/6	66–96	*CADPS2, TH, IL1B, GPNMB, HSP90AA1, CD44, S100B*	[120]
Substancia nigra	52/60	61–92	*SLC18A2, VMAT2, NEFL, DDC*	[157]

* The studies included in this table were selected in a targeted manner based on methodological rigor and biological relevance. We considered only articles published from 2015 onwards that used human samples (post-mortem tissue, peripheral blood, PBMCs, CSF, iPSC-derived cells, or single-cell/single-nucleus profiles) and performed gene expression analysis (bulk or single-cell/single-nucleus RNA-seq, microarrays, or targeted panels) with explicit identification of DEGs between relevant groups of interest (e.g., PD vs. controls), and that reported the number of participants and demographic data either in the main text or Supplementary Material. We excluded studies based exclusively on miRNAs, circRNAs, or other non-coding RNAs without corresponding mRNA DEGs, purely genetic analyses and genome-wide association studies (GWAS), narrative reviews, commentaries, resource/data descriptors without case–control expression contrasts, as well as purely bioinformatic re-analyses that did not report clearly extractable DEG lists in a standardized manner.

## Data Availability

No new raw data were generated in this study. The contributions presented in this study are included in the article/Supplementary Material.

## Supplementary Materials

The following supporting information can be downloaded at: https://www.mdpi.com/article/10.3390/cimb48030254/s1.

## Abbreviations

3-NT	3-nitrotyrosine
α-Syn	α-Synuclein
A2A	Adenosine A2A receptor
ACO2	Aconitase 2 (mitochondrial aconitase)
ADLs	Activities of Daily Living
BBB	Blood–brain barrier
BCAA	Branched-chain amino acids
CKD	Chronic kidney disease
CNS	Central nervous system
COMT	Catechol-O-methyltransferase
Cav1.3	L-type voltage-gated calcium channel CaV1.3
CSF	Cerebrospinal fluid
D2/D3	Dopamine D2/D3 receptors
DA	Dopamine
DBS	Deep brain stimulation
DEGs	Differentially expressed genes
DMT1	Divalent Metal Transporter 1
DRG	Downregulated genes
ER	Endoplasmic reticulum
ETC	Electron transport chain
FAO	Fatty acid oxidation
FDG-PET	Fluorodeoxyglucose positron emission tomography
FOG	Freezing of gait
GBA	Glucocerebrosidase gene
GCase	Glucocerebrosidase
GLUT1/3	Glucose transporters 1/3
GPi	*Globus pallidus internus*
GSH	Reduced glutathione
GWAS	Genome-wide association studies
iNOS	Inducible nitric oxide synthase
IDH3G	Isocitrate dehydrogenase (NAD^+^) subunit gamma
KGDHC	α-Ketoglutarate dehydrogenase complex
LCIG	Levodopa/carbidopa intestinal gel
LRRK2	Leucine-rich repeat kinase 2
MPTP	1-methyl-4-phenyl-1,2,3,6-tetrahydropyridine
MAO-B	Monoamine oxidase B
MAOIs	Monoamine oxidase inhibitors
MCU	Mitochondrial calcium uniporter
MDA	Malondialdehyde
MDH2	Malate dehydrogenase 2
mPTP	Mitochondrial permeability transition pore
MRI	Magnetic resonance imaging
NADH	Nicotinamide adenine dinucleotide (reduced form)
NADPH	Nicotinamide adenine dinucleotide phosphate (reduced form)
NMDA	N-methyl-D-aspartate (receptor)
NLR	Neutrophil-to-lymphocyte ratio
NLRP3	NOD-, LRR- and pyrin domain-containing protein 3
OGDH	Oxoglutarate dehydrogenase
OPG	Osteoprotegerin
OXPHOS	Oxidative phosphorylation
Parkin	E3 ubiquitin ligase Parkin
PBMCs	Peripheral Blood Mononuclear Cells
PEG-J	Percutaneous endoscopic gastrojejunostomy
*PINK1*	PTEN-induced kinase 1
PD	Parkinson’s disease
PPP	Pentose phosphate pathway
PRR	Pattern Recognition Receptors
QoL	Quality of life
RNA-Seq	RNA sequencing
ROS	Reactive oxygen species
SCFA	Short-chain fatty acids
scRNA-Seq	Single-cell RNA sequencing
SDH	Succinate dehydrogenase
SNpc	*Substantia nigra pars compacta*
SSRIs	Selective Serotonin Reuptake Inhibitors
STN	Subthalamic nucleus
TCA	Tricarboxylic acid cycle
TNF-α	Tumor Necrosis Factor alpha
UPS	Ubiquitin–proteasome system
UPDRS	Unified Parkinson’s Disease Rating Scale
URG	Upregulated genes
